# *“Going vaccine hunting”*: Multilevel influences on COVID-19 vaccination among racialized sexual and gender minority adults—a qualitative study

**DOI:** 10.1080/21645515.2023.2301189

**Published:** 2024-02-12

**Authors:** Peter A. Newman, Duy Anh Dinh, Notisha Massaquoi, Charmaine C. Williams, Ashley Lacombe-Duncan, Suchon Tepjan, Thabani Nyoni

**Affiliations:** a Factor-Inwentash Faculty of Social Work, University of Toronto, Toronto, Ontario, Canada; b Faculty of Health Sciences, Queen’s University, Kingston, Ontario, Canada; c Department of Health and Society, University of Toronto, Scarborough, Ontario, Canada; d School of Social Work, University of Michigan, Ann Arbor, MI, USA; e VOICES-Thailand Foundation, Chiang Mai, Thailand; f School of Social Work, Faculty of Health, Dalhousie University, Halifax, Nova Scotia, Canada

**Keywords:** COVID-19, vaccination, vaccine hesitancy, vaccine acceptance, vaccine equity, structural determinants of health, medical mistrust, racial/ethnic minority, LGBTQ, Canada

## Abstract

High levels of COVID-19 vaccine hesitancy have been reported among Black and Latinx populations, with lower vaccination coverage among racialized versus White sexual and gender minorities. We examined multilevel contexts that influence COVID-19 vaccine uptake, barriers to vaccination, and vaccine hesitancy among predominantly racialized sexual and gender minority individuals. Semi-structured online interviews explored perspectives and experiences around COVID-19 vaccination. Interviews were recorded, transcribed, uploaded into ATLAS.ti, and reviewed using thematic analysis. Among 40 participants (mean age, 29.0 years [SD, 9.6]), all identified as sexual and/or gender minority, 82.5% of whom were racialized. COVID-19 vaccination experiences were dominated by structural barriers: systemic racism, transphobia and homophobia in healthcare and government/public health institutions; limited availability of vaccination/appointments in vulnerable neighborhoods; absence of culturally-tailored and multi-language information; lack of digital/internet access; and prohibitive indirect costs of vaccination. Vaccine hesitancy reflected in uncertainties about a novel vaccine amid conflicting information and institutional mistrust was integrally linked to structural factors. Findings suggest that the uncritical application of “vaccine hesitancy” to unilaterally explain undervaccination among marginalized populations risks conflating structural and institutional barriers with individual-level psychological factors, in effect placing the onus on those most disenfranchised to overcome societal and institutional processes of marginalization. Rather, disaggregating structural determinants of vaccination availability, access, and institutional stigma and mistrust from individual attitudes and decision-making that reflect vaccine hesitancy, may support 1) evidence-informed interventions to mitigate structural barriers in access to vaccination, and 2) culturally-informed approaches to address decisional ambivalence in the context of structural homophobia, transphobia, and racism.

## Introduction

Vaccines are a life-saving intervention. Before the COVID-19 pandemic, childhood vaccination prevented an estimated 2–3 million children’s deaths globally each year.^1^ In the pandemic context, vaccination resulted in an estimated 63% (14.4–19.8 million) reduction in deaths globally in the first year of COVID-19 alone.^2,3^ Vaccine booster doses^4^ and bivalent vaccine boosters^5^ have further reduced COVID-19-associated hospitalization and death. Nevertheless, disparities in COVID-19 morbidity and mortality among marginalized populations, as well as lower rates of COVID-19 vaccination, suggest that the science and practices guiding vaccine dissemination continue to lag those that underlie vaccine development.

The COVID-19 pandemic exerted a disproportionate impact on marginalized communities. Higher rates of infection and mortality among racial and ethnic minority populations^6–11^ and greater vulnerability among sexual and gender minority populations^12,13^ documented in Canada and the United States (U.S.) indicate a greater need for COVID-19 vaccination. Nevertheless, several studies indicate higher levels of vaccine hesitancy and undervaccination among marginalized populations, along with rampant misinformation and the politicization of COVID-19 and measures to control it.^14–16^ Defined as a delay in acceptance or refusal of vaccination despite the availability of vaccination services,^17^ vaccine hesitancy has been identified as a substantial threat to global health.^1^ Greater COVID-19 vaccine hesitancy has been described among Black and other racialized populations in Canada^18–20^ and the U.S.^21–24^ in contrast to White populations, although with inconsistent findings.^18^ Limited data indicate greater vaccine hesitancy and lower vaccination coverage among Black adults across all categories of minoritized sexual orientation and gender identities^25–27^ and less confidence about vaccine safety among transgender and gender diverse adults than among cisgender adults.^26^

Critiques of the term “vaccine hesitancy” articulated both prior to^17,28–30^ and during the COVID-19 pandemic^31^ describe it as imprecise, ambiguous in its application, and overly behavioral, with calls to address the complexity and context-specific nature of vaccine hesitancy. Nevertheless, amid burgeoning research on COVID-19 vaccination, the vast majority of studies attribute undervaccination among marginalized populations predominantly or wholly to vaccine hesitancy, with scant research exploring the perspectives and lived realities of sexual and gender minority and racialized populations. The purpose of this study is to examine multilevel factors that contribute to COVID-19 undervaccination and vaccine hesitancy, including structural, social and community, and individual factors, among individuals from sexual and gender minority and racialized populations. Such evidence is critical to supporting the equitable allocation and effective dissemination of COVID-19 vaccines in North America and globally.^32^

## Methods

### Setting and participant selection

Participants were sampled from a cohort of lesbian, gay, bisexual, transgender, nonbinary, queer and other (LGBTQ+) individuals, predominantly racial/ethnic minority, who had completed an online survey about COVID-19 pandemic-related knowledge, psychological distress, and coping from March to November 2021. Inclusion criteria were self-identifying as a sexual and/or gender minority person, being age 18 years or older, and resident in the Greater Toronto and Hamilton Area (GTHA) in Ontario, Canada. In the present study, we focused on LGBTQ-identified adults, and those at sexual/gender minority and ethnic/racial minority demographic intersections, due to the scant data regarding their experiences of the pandemic^25–27^ and COVID-19 vaccination^32,33^ despite heightened vulnerability, along with global evidence of challenges to vaccine acceptance^31^ and equity among marginalized populations.^32^ To that end, we purposively sampled individuals from the larger survey cohort with the goal of recruiting a racially and ethnically diverse, and sexually and gender diverse, sample of LGBTQ+ individuals.

All interviews were conducted from June to September 2022. Initial COVID-19 vaccination in the GTHA was rolled out incrementally from early- to mid-2021, first to adults >70 years-old, then to adults >50 years-old and those with certain health conditions, and finally made available to the general population from July 2021. During the period of the interviews, COVID-19 vaccine booster doses were being administered in the GTHA.

### Data collection

A semi-structured interview guide was developed based on published reviews and studies of vaccination and vaccine hesitancy^14,34,35^ and informed by a social ecological framework.^36^ The latter conceptualizes multilevel influences on health and wellbeing, including structural, social and community, and individual levels.^36,37^ Questions and probes were also guided by the expertise of our community-university research team with extensive experience working with sexual and gender minority and racialized populations in the GTHA.^38^

In-depth semi-structured interviews were conducted online by trained research assistants, all of whom were either from the focal communities or had experience working with these communities, with biweekly team meetings for supervision and debriefing. Open-ended questions explored perspectives and experiences around COVID-19 vaccination, including individual-, social- and structural-level concerns (see Supplemental File 1). The study was approved by the Research Ethics Board of the University of Toronto (#39769).

### Data analysis

All interviews were digitally recorded, transcribed verbatim by the research team, uploaded into ATLAS.ti qualitative data management software for groups (ATLAS.ti Scientific Software Development GmbH, Berlin, Germany), and then analyzed using an interpretive process of thematic analysis.^39^ Ongoing research team meetings included training in ATLAS.ti and co-development of an initial codebook based on the interview guide. The codebook was first applied deductively by 2 independent coders to the same transcripts, then reviewed in team meetings and revised iteratively to include inductively identified emergent codes. After 2 rounds of independent coding, consensus was reached, and subsequent transcripts were coded individually. Coded text was then reviewed and critically assessed using generative coding, which involved working the tensions between deductive application of codes to formulate subthemes within social ecological levels, and inductive processes of critically reflecting on, re-defining, merging, and parsing codes to formulate subthemes and their broader thematic categorization.^39^ This enabled re-shaping of themes to enrich understanding of COVID-19 vaccination and “vaccine hesitancy” from the perspectives of racialized sexual and gender minority communities.

Deidentified interview transcripts were encrypted and stored securely on ATLAS.ti Web and shared by the research manager through password-protected access with the designated coder(s) and lead study investigators. Quality assurance was maintained throughout the research process by means of ongoing researcher reflexivity, transparency in decision-making, communication with the research team, and upholding good ethical practice.^40,41^

## Results

Forty participants were interviewed, with a mean duration of 47 minutes (SD, 13). Participants’ mean age was 29.0 years (SD, 9.6). All identified as sexual and/or gender minority: 47.5% (*n* = 19) were cisgender lesbian, bisexual, or pansexual women, 32.5% (*n* = 13) transgender people (7 trans women, 6 trans men), 12.5% (*n* = 5) nonbinary, and 7.5% (*n* = 3) cisgender gay men. The majority identified as people of color: 42.5% (*n* = 17) were Black, 30.0% (*n* = 12) Asian, 7.5% (*n* = 3) Latinx, 17.5% (*n* = 7) White, and 2.5% (*n* = 1) multiracial. Over two-thirds (67.5%, *n* = 27) of participants were single, with 12.5% (*n* = 5) married and 20.0% (*n* = 8) partnered (to a same- or opposite-sex partner). Just over half (52.5%; *n* = 21) had college-degree education. Equal numbers were employed (*n* = 15; 37.5%) and unemployed (*n* = 15; 37.5%), 17.5% (*n* = 7) students and 7.5% (*n* = 3) accessing disability income support. Most (*n* = 35; 87.5%) reported having government and/or private health insurance coverage. Participants’ demographic characteristics are shown in Table 1.

**Table 1.** Participant demographics from in-depth interviews with racially/ethnically diverse sexual and gender minority people in Toronto, Canada, 2022 (n *=* 40).

PID	Age (yrs)	Race/Ethnicity	Gender identity	Sexuality	Currently in relationship	Education	Employment status
1	24	White	Cisgender woman	Lesbian	No	College	Disability
2	23	East Asian/White	Cisgender man	Gay	No	College	Part-time
3	23	South Asian	Cisgender man	Gay	No	College	Part-time
4	19	East Asian	Cisgender woman	Two-spirit	No	High school	Student
5	24	Black/Caribbean	Cisgender woman	Lesbian	No	College	Part-time
6	23	Black/Caribbean	Cisgender woman	Bisexual	No	College	Part-time
7	24	Black/Caribbean	Cisgender man	Gay	No	College	Full-time
8	24	White	Cisgender woman	Pansexual	No	College	Unemployed
9	23	East Asian	Cisgender woman	Two-spirit	No	College	Part-time
10	34	White	Transgender woman	–	Yes	High school	Unemployed
11	19	White	Transgender man	Other	No	High school	Student
12	42	Black/Caribbean	Transgender woman	Heterosexual	Yes	College	Unemployed
13	31	Black/African	Transgender woman	Pansexual	No	High school	Disability
14	36	Black/Caribbean	Transgender woman	Other	Yes	High school	Unemployed
15	28	Black/Caribbean	Transgender man	Other	Yes	College	Part-time
16	19	Southeast Asian	Transgender man	Other	No	High school	Student
17	38	Latinx	Transgender man	–	Yes	High school	Full-time
18	23	South Asian	Transgender man	Pansexual	Yes	College	Unemployed
19	28	Black/Caribbean	Transgender woman	–	No	High school	Unemployed
20	23	White	Transgender woman	–	No	Elementary	Disability
21	52	White	Transgender man	–	Yes	High school	Unemployed
22	25	White	Transgender woman	–	No	College	Unemployed
23	24	Black/Caribbean	Cisgender woman	Lesbian	No	College	Student
24	50	Black/Caribbean	Cisgender woman	Other	Yes	High school	Unemployed
25	24	Black/Caribbean	Cisgender woman	Two-Spirit	No	High school	Unemployed
26	30	Black/Caribbean	Cisgender woman	Pansexual	No	High school	Part-time
27	31	East Asian	Nonbinary (AFAB)	Lesbian	No	College	Unemployed
28	25	Southeast Asian	Nonbinary (AFAB)	Pansexual	No	High school	Part-time
29	25	Black/African	Nonbinary (AFAB)	Two-spirit	No	College	Part-time
30	37	Latinx	Cisgender woman	Other	Yes	High school	Student
31	25	South Asian	Cisgender woman	Two-spirit	No	College	Full-time
32	19	Southeast Asian	Cisgender woman	Lesbian	Yes	College	Part-time
33	32	East Asian	Cisgender woman	Lesbian	No	College	Unemployed
34	27	South Asian	Cisgender woman	Lesbian	No	High school	Unemployed
35	32	Black/Caribbean	Cisgender woman	Pansexual	No	High school	Unemployed
36	58	Black/Caribbean	Cisgender woman	Heterosexual	No	High school	Part-time
37	29	East Asian	Cisgender woman	Lesbian	Yes	College	Student
38	48	Black/African	Cisgender woman	Bisexual	Yes	College	Part-time
39	18	Latinx	Nonbinary (AFAB)	Pansexual	Yes	High school	Student
40	22	Black/Caribbean	Nonbinary (AFAB)	Two-spirit	No	College	Unemployed

AFAB, assigned female sex at birth.

We defer to participant self-identifications but acknowledge the problematical use of the term “two-spirit” by non-indigenous people to denote gender identity or sexual orientation.

**Table 2.** Structural determinants of COVID-19 vaccination.

Subtheme	Illustrative Quotations
Physical unavailability and inaccessibility	*“ … at one point we were kind of like going vaccine hunting because I wasn’t … living in a postal code where we were eligible for it yet and so we were going to other clinics to try and see if we can get” (P2, 23 y/o, East Asian/White, cisgender man, gay)* *“There were absolutely not enough pop-up clinics, especially within racialized areas” (P3, 23 y/o, South Asian, cisgender man, gay)*
Information access	*”…education was lacking. Awareness is lacking. I don’t really blame the people, I blame the systems in place” (P3, 23 y/o, South Asian, cisgender man, gay)* *“I definitely know like other grannies who came to Canada like a long time ago, and they mainly stay in the East Asian or the Chinese community and they can’t really speak English. … sometimes it was hard for them to even just get the notification or understand the policy. Second, understanding where do you even get the vaccine? And when they get there, there’s no translation service, so they can’t even communicate.” (P9, 23 y/o, East Asian, cisgender woman, two-spirit)*
Digital/internet access	*“I had to sit on the portal and refresh for days and days to get an appointment” (P8, 24 y/o, White cisgender woman, pansexual)* *“I think the government website … for some reason seems to be the most inaccessible; these are websites that have important information, and you would think that website would be developed with that in mind, you know?” (P6, 23 y/o, Black/Caribbean, cisgender woman, bisexual)*
Insurance and immigration	*“ … my partner who was going through the immigration process and at the time didn’t have an OHIP (government health insurance) card had a harder time accessing something as basic as a vaccination… a lot of those people were left out. … when there were requirements made, asking for the copy of your status. (P8, 24 y/o, White, cisgender woman, pansexual)* *“I don’t have like a third or a booster just because I changed provinces and there’s some issue with accessing it right now.” (P27, 31 y/o, East Asian, nonbinary [AFAB], lesbian)*
Indirect vaccination costs	*”…for a lot of people who’ve been on (welfare), just being able to afford the bus fare to get the vaccine was challenging” (P1, 24 y/o, White, cisgender woman, lesbian)* *”…we stood in the rain for 6 hours so that she can get one. … because Pfizer was in high demand” (P32, 19 y/o, Southeast Asian, cisgender woman, lesbian)*
Structural racism, transphobia, and homophobia	*“When you go back in your history and you see the studies that they did on Black people, and what they did to the Africans, you are gonna be very hesitant to take any vaccine” (P14, 36 y/o, Black/Caribbean, transgender woman)* *“Folks are normally misgendered and sometimes it can be a very toxic space…to be in” (P23, 24 y/o, Black/Caribbean, cisgender woman, lesbian)*

Findings are organized into three overarching themes comprising structural, social and community, and individual determinants of COVID-19 vaccination, each with various subthemes. Quotations illustrating subthemes are presented in tables under each theme and in the text, with labels indicating participant ID, age, race/ethnicity, gender identity and/or sexual orientation.

### Structural determinants of COVID-19 vaccination

The structural context of COVID-19 vaccination was characterized by various dimensions of vaccine inaccessibility for marginalized populations (see Table 2).

*Physical Unavailability and Inaccessibility: “Going vaccine hunting”*

Access to COVID-19 vaccination during the pandemic was reported to be a function of geography, particularly one’s zip code, with different risk levels ascribed by public health departments based on one’s neighborhood in the Greater Toronto and Hamilton Area: “ … *because the postcode that I’m living at … they evaluated as a low-risk area. So, all the people in my postcode got the vaccine really, really late*” (P33, 32 y/o, East Asian, cisgender woman, lesbian). Access gradually became easier as the pandemic progressed: *“Towards the end with vaccine distribution … when I came back to Toronto, how much easier it was to get vaccination here”* (P29, 25 y/o, Black/African, nonbinary [assigned female sex at birth (AFAB)], two-spirit). Expedited access to vaccines was reported by those who worked or volunteered in healthcare facilities and those with relatives working in these facilities.

Amid constrained access, participants described various forms of “vaccine hunting:” “ … *at one point we were kind of like going vaccine hunting because I* *wasn’t* *… living in a* *postal code where we were eligible for it yet* … ” (P2, 23 y/o, East Asian/White, cisgender man, gay). Another participant described informal community generated efforts: *“… there was a thing called Vaccine Hunters where people, they said, we’re gonna have this responsibility on ourselves to tell people which clinics had the vaccines”* (P6, 23 y/o, Black/Caribbean, cisgender woman, bisexual).

Several participants reported limited access as a function of having only one hospital in their local community that provided COVID-19 vaccination. Pop-up clinics were reported to be more geographically dispersed, but less prevalent in racialized than in White communities:

There were absolutely not enough pop-up clinics, especially within racialized areas. … within Toronto, where there are white people, there were so many pop-ups; but in places like Brampton, Mississauga or low-income areas, there were not enough pop-ups. (P3, 23 y/o, South Asian, cisgender man, gay)

The location of racialized and sexual/gender minority people in underserviced and deprioritized neighborhoods exacerbated challenges to getting vaccinated. Specific barriers in access were reported for older adults and those with mobility challenges, for example: “*… it’s really hard for her to even make it outside because she has a medical condition. And she’s been calling places and they haven’t been accessible to her”* (P23, 24 y/o, Black/Caribbean, cisgender woman, lesbian).

*Information Access: “I don’t … blame the people, I blame the systems in place”*

Challenges around lack of access to clear information about COVID-19 and vaccination were described, including *“the rules and regulations and the requirements for vaccines … It was just really, really complicated”* (P28, 25 y/o, Southeast Asian, nonbinary [AFAB], pansexual). Frustrations about limited vaccine supply and access were compounded by this uncertainty and the perceived erratic nature and sources of information: “ *… there were just so few places that were doing it; and then when they were doing it, they would like kind of just pop up out of nowhere”* (P20, 23 y/o, White, transgender woman).

Access to information was further constricted due to lack of availability of translations in multiple languages about COVID-19, vaccines, and where and how to access vaccination. Participants also reported an absence of translators at vaccination sites. Systemic barriers in access to accurate information for those not fluent in English amplified risks of relying on misinformation, *“ … because they hear different comments about the vaccine from different places, and some of them think it is dangerous to get the vaccine”* (P33, 32 y/o, East Asian, cisgender woman, lesbian).

*Digital/Internet Access: “Sit on the portal and refresh for days”*

Participants described challenges around digital access and digital literacy: *“It was very hard to get an appointment”* (P8, 24 y/o, White cisgender woman, pansexual). Challenges were exacerbated for older adults and those whose primary language was not English, as well for those without access to IOT (Internet of Things) devices (e.g., smartphones, laptops). Several younger participants reported seeking assistance for older adult parents and neighbors to navigate online appointments and others reported booking the appointments for them: *“She doesn’t really know how to use technology, so I’ve been trying my best to find someone who can come in and help her with that”* (P23, 24 y/o, Black/Caribbean, cisgender woman, lesbian).

*Insurance and Immigration: “A lot of … people were left out”*

Despite universal access to healthcare in Canada, participants who identified as immigrants and newcomers recounted that *“a lot of those people were left out* *… when there were requirements made, asking for the copy of your status”* (P8, 24 y/o, White, cisgender woman, pansexual). Challenges were exacerbated for transgender immigrants whose processes often take longer due to having documents from their country of origin with only their birth name:

I’m a trans woman and I immigrated in 2019, just before the pandemic really hit. I was very desperate to get one for a really long time, but because I did not have OHIP (provincial insurance), I could not get my first vaccine until August last year [2022]. (P22, 25 y/o, White, transgender woman)

Officially, individuals were not required to show a health card to be vaccinated at government venues, however many government websites for booking appointments required inputting a health card number, effectively requiring one to walk in. Some alternate sites (i.e., pharmacies, etc.) reportedly requested that people show their health cards on site; challenging that inappropriate demand was a more daunting prospect for immigrants, especially those not fluent in English and/or marginalized by virtue of their race/ethnicity or gender identity.

*Indirect Vaccination Costs: “I can’t really take the time off work”*

Although there was no out-of-pocket cost for vaccination, many indirect costs emerged as barriers: costs of transportation to sites outside one’s neighborhood, missing work without paid sick days, childcare costs, and long wait times. A White trans man reported, *“It’s pretty hard. I could get one tomorrow, but I can’t really take the time off work with the hours offered to me”* (P21, 52 y/o, White, transgender man). A Latinx trans man explained: *“A lot of folks in our community, they couldn’t, or they needed child-minding but they didn’t have the funds; there’s so many different accessibility issues”* (P17, 38 y/o, Latinx, transgender man). Although motivated to seek vaccination as soon as they were eligible, many reported excessive wait times:

I would go to the pop-up clinics, cos’ I had an appointment but it was like four months away and I didn’t wanna wait. So, I would call public clinics and they would say, ‘yeah, we have Pfizer’. And you would go and wait like six hours in line, and it would be your turn. (P35, 32 y/o, Black/Caribbean, cisgender woman, pansexual)

*Structural Racism, Transphobia and Homophobia: “The healthcare system is … wicked and cruel against Blacks”*

The impacts of structural racism in healthcare systems, government public health, and the pharmaceutical industry were reflected in participants’ references to histories of unethical medical experimentation, disparities in access to care, and ongoing experiences of racial stigma and discrimination. A Black/African transgender woman evoked both historical and present-day manifestations of structural racism in reverting from the present to the past and then back to the present moment: *“ … the healthcare system is, was, is wicked and cruel against Blacks*” (P13, 31 y/o, Black/African, transgender woman, pansexual). A Black/Caribbean transgender woman articulated the impact of historical injustices and ongoing inequity due to structural racism: *“before they give it to the rich, they perfected it on the poor”* (P14, 36 y/o, Black/Caribbean, transgender woman).

Several transgender and gender diverse individuals articulated barriers due to “deadnaming”—i.e., being referred to by a name assigned in infancy in cases where they had rejected that name^42^—and more generally being misgendered in unfamiliar healthcare settings and vaccination sites: *“As a trans person, it is really awkward to go and then have your name card and then they’re like, ‘oh yes, Deadname.’”* (P16, 19 y/o, Southeast Asian, transgender man). A White trans woman explained, “*I know a lot of trans people that didn’t get their vaccination because they were either afraid of being deadnamed, misgendered, or just their own gender dysphoria induced agoraphobia …* ” (P20, 23 y/o, White, transgender woman). A Black/Caribbean trans woman summed up concerns about seeking vaccination in places deemed unsafe: *“I could tell you right now, they did not consider trans people”* (P19, 28 y/o, Black/Caribbean, transgender woman).

Structural homophobia and transphobia were also described as barriers to vaccination among sexual minorities: *“I think the wider queer and BIPOC [Black, Indigenous, and People of Color] community does have reason to be skeptical of government health stuff”* (P22, 25 y/o, White, transgender woman). An Asian sexual minority woman, evoking her intersectional marginalized identities, reported “*there was a lot of mistrust in a lot of folks in the communities that I’m part of and the identities that I share*” (P27, 31 y/o, East Asian, nonbinary [AFAB], lesbian). Another Asian sexual minority woman’s narrative echoed this theme: *“it just further confirmed my own fears … not fears, my own doubts around it, where the healthcare system has failed me in so many ways. Why would I trust a vaccine that is relatively new?”* (P34, 27 y/o, South Asian, cisgender woman, lesbian).

### Social and community determinants of COVID-19 vaccination

The social and community context for COVID-19 vaccination was largely reported in positive influences on vaccine uptake, comprising interpersonal trust in vaccine recommenders, family and community influences, and altruistic motivations (see Table 3).

**Table 3.** Social and community determinants of COVID-19 vaccination.

Subtheme	Illustrative Quotations
Trust in vaccine recommender	*“A family doctor, because they have the medical experience…and I trust them” (P1, 24 y/o, White, cisgender woman, lesbian)* *“…not the government health professionals, the actual doctors – I listened” (P21, 52 y/o, White, transgender man)*
Family and community influence	*“Seeing folks in my community doing it…helped me be like, okay, I’m gonna do this as well” (P27, 31 y/o, East Asian, nonbinary [AFAB], lesbian)* *“Thankfully my family, like they don’t understand the science too much, but being a student in the sciences, when I told them they were never against any vaccine. Like they got me and my sister vaccinated for everything when we were kids.” (P40, 22 y/o, Black/Caribbean, nonbinary [AFAB], two-spirit)*
Altruism	*“We don’t mind, we want to keep our community safe” (P38, 48 y/o, Black/African, cisgender woman, bisexual)* *“To protect my family from infection, to protect myself from infection, to limit the spread of COVID-19.” (P3, 23 y/o, South Asian, cisgender man, gay)*

*Trust in Vaccine Recommender: “I trust that they have better data than my dad!”*

Trusted sources of COVID-19 vaccine recommendations differed across participants, however several indicated a preference for a doctor or a healthcare provider from a local clinic: *“Doctors, because they are trained; they do this research, they are educated … so I would trust doctors the most”* (P3, 23 y/o, South Asian, cisgender man, gay). Participants specifically differentiated frontline physicians from government public health officials: *“Whatever is recommended by the health professionals – not the government health professionals, the actual doctors – I listened”* (P21, 52 y/o, White, transgender man). However, a few participants voiced preferences for government public health recommendations, even while noting trepidations:

I actually do trust the FDA and the CDC generally in terms of vaccination recommendations. They have a sketchy history, especially for Black/People of Color communities in the States. I hope those days are gone; but I trust that they have better data than my dad! (P22, 25 y/o, White, transgender woman)

Notably, this favoring of information from public health authorities was not voiced by any Black participants. One participant invoked a caveat in according greater influence to a government recommendation – if presented with evidence that *“they* *took* *the vaccine … after that, I feel ok, yes … I can take it”* (P37, 29 y/o, East Asian, cisgender woman, lesbian).

*Family and Community Influence: “Seeing folks in my community doing it”*

A range of influences were described across partners and family, and local community: *“ … it was less like asking a medical professional or a vaccine professional; it was more like speaking to folks in your community and seeing how [they felt]”* (P5, 24 y/o, Black/Caribbean, cisgender woman, lesbian). Influence was also described within the family: *“me and my husband, we had a discussion”* (P12, 42 y/o, Black/Caribbean, transgender woman, heterosexual). A young person explained: *“ … my family, like they don’t understand the science too much, but being a student in the sciences, when I told them, they were never against any vaccine”* (P40, 22 y/o, Black/Caribbean, nonbinary [AFAB], two-spirit).

*Altruistic Motivations: “We don’t mind, we want to keep our community safe”*

At the social and community level, altruistic motivations exerted a substantial and sometimes primary influence on vaccination based on a desire to protect one’s family, especially older relatives, and one’s broader community, including vulnerable individuals. A Black cisgender gay man explained: *“My grandparents, honestly; my grandma’s my best friend. I honestly do it just for her”* (P7, 24 y/o, Black/Caribbean, cisgender man, gay). Similarly, a Southeast Asian trans man noted, “*I think some stuff that influenced me to get vaccinated was that my parents were super old … ”* (P16, 19 y/o, Southeast Asian, transgender man). Some participants specifically described benefits based on herd immunity: “*I wanted to stay safe, I wanted to keep others safe. I wanted to do my part for the community because I know that herd immunity is so important…”* (P1, 24 y/o, White, cisgender woman, lesbian). Others similarly described blanket acceptance of COVID-19 vaccination based on a primary motivation to protect their family and community.

### Individual-level determinants of COVID-19 vaccination

The individual-level context for COVID-19 vaccination was defined by a theme of vaccine hesitancy, in addition to the perceived threat of COVID-19 and personally researching available information (see Table 4).

**Table 4.** Individual-level determinants of COVID-19 vaccination.

Subtheme	Illustrative Quotations
Vaccine hesitancy	*“I didn’t want to take it at the very beginning. … it showed up too fast. I don’t know. Everyone doesn’t know. Even those experts don’t know if there are unknown side effects” (P37, 29 y/o, East Asian, cisgender woman, lesbian)* *“As of right now, I still feel very strongly like, no, this isn’t for me! … as a Black person, the healthcare system doesn’t care about me” (P34, 27 y/o, South Asian, cisgender woman, lesbian)*
Perceived threat of COVID-19	*“I was really scared because I saw a lot of people dying from it” (P25, 24 y/o, Black/Caribbean, cisgender woman, two-spirit)* *“After looking at how many people kind of died from it and how ill people seriously got from getting COVID, I opted to get the vaccine because I would rather have gotten the vaccine instead of gotten COVID.” (P35, 32 y/o, Black/Caribbean, cisgender woman, pansexual)*
Personal research/scientific literacy	*“I trust papers way more than people specifically. So yeah, I trust clinical trials” (P9, 23 y/o, East Asian, cisgender woman, two-spirit)* *“I followed the science behind vaccines for years before the pandemic, so I really wasn’t all that concerned.” (P1, 24 y/o, White, cisgender woman, lesbian)*

*Vaccine Hesitancy: “A brand new vaccine and we don’t know the long-term effects”*

A spectrum of concerns emerged from initial uncertainty, ambivalence, or confusion, to initial refusal, to more enduring resistance to COVID-19 vaccination. Several participants expressed initial caution and concern, even specifically invoking “hesitancy:” *“I was very hesitant to go and get it because it’s a brand new vaccine and we don’t know the long term effects of it”* (P35, 32 y/o, Black/Caribbean, cisgender woman, pansexual). As another participant reported: *“So, I* *will just wait* *… my point is I* *don’t, hate it, but I* *did hesitate at the beginning”* (P37, 29 y/o, East Asian, cisgender woman, lesbian). Other narratives directly invoked ambivalence, in one case based on medical uncertainty about recommendations for pregnant people:

I had to make the really tough choice of choosing whether or not to get vaccinated and it was potentially, … they weren’t sure if it was dangerous or enough being as I was pregnant. (P8, 24 y/o, White, cisgender woman, pansexual)

Participants reported the need for more information, uncertainty about conflicting accounts and whom to trust—*“the uncertainty of everything, just like not knowing … I don’t know who to believe”* (P18, 23 y/o, South Asian transgender man, pansexual)—as well as seeking particular sources of COVID-19 vaccine recommendation: *“ … nervous about the vaccines … so I did wait a little bit just before getting them, so I could talk to my doctor about it”* (P11, 19 y/o, White, transgender man). Some participants articulated the need for time to feel confident in their own decision:

So, I need to make sure that I am a hundred percent sure that this is something I wanna do. Because if it doesn’t work out, I don’t wanna feel like I just did it because I was told to do it. I just wanna feel like … I’m secure in my decision. (P34, 27 y/o, South Asian, cisgender woman, lesbian)

A few participant narratives illustrate what has been referred to as the ‘resistant’ end of the vaccine hesitancy spectrum. Their accounts also complicate conceptualizations of vaccine resistance in their being pervaded with experiences of structural racism. One participant decried exhortations to take booster doses, which exacerbated her hesitancy around COVID-19 vaccination in the context of mistrust of a healthcare system perceived as failing to consider the needs and experiences of people of color—as reflected in the intentional shift from “fears” to “doubts:”

Why do you need so many booster shots? If it worked, it worked! Oh, it doesn’t work? Your immune system is still shaky? Like it just confirmed my own fears, not fears, my own doubts around it, where the healthcare system has failed me in so many ways … . (P34, 27 y/o, South Asian, cisgender woman, lesbian)

Another participant framed her vaccine stance as being “pro-choice,” however subsequently revealing frustration at the frequent suggestion that if only a Black doctor talked to her, she might then get vaccinated:

I feel like I’m not against it, but I’m also pro-choice. I feel like get it if you want to get it, don’t get it if you don’t want to. I’ve thought about it, I’ve spoken to multiple people about it. … I’ve gotten questions like, ‘does it help if a Black doctor tells you to get it?’ It doesn’t. It doesn’t f–king help. I really don’t care who tells me to get it, ‘cause I’m not! (P5, 24 y/o, Black/Caribbean, cisgender woman, lesbian)

This narrative depicts a Black woman’s experiences of perceived coercion in the healthcare system and the reductionistic assumption that individual interaction with a doctor of one’s own ethnicity will automatically dispel the context of structural racism.

*Perceived Threat of COVID-19: “I saw a lot of people dying from it”*

Several participants recounted a context of fear reflected in the media and “scary stories” from coworkers and their local communities. A Latinx trans man described hearing from a young person at his work that they had contracted COVID-19 and unwittingly *“brought it home and my whole family got sick, and my dad died of it”* (P17, 38 y/o, Latinx, transgender man). Similarly, a Black/Caribbean cisgender woman explained, *“I think I was really scared because I saw a lot of people dying from it, like in the news and nearby hospitals … ”* (P25, 24 y/o, Black/Caribbean, cisgender woman, two-spirit). Collectively, these were described as motivations for getting vaccinated. As another Black/Caribbean woman surmised, “*I opted to get the vaccine because I would rather have gotten the vaccine instead of gotten COVID*” (P35, 32 y/o, Black/Caribbean, cisgender woman, pansexual).

*Personal Research/Scientific Literacy: “I trust papers way more than people”*

Some participants described variations of the premise that, *“I took it upon myself to do the research”* (P13, 31 y/o, Black African, transgender woman, pansexual). Inherent in this was trust in one’s own ability to locate and evaluate the evidence more so than others’ recommendations: *“ … so far, compared to any other person, I trust papers way more than people … ”* (P9, 23 y/o, East Asian, cisgender woman, two-spirit). Another participant indicated, *“I did some research … it was the government website”* (P6, 23 y/o, Black/Caribbean, cisgender woman, bisexual). Thus some, particularly college-educated, participants accessed sources of scientific evidence to make an informed decision about COVID-19 vaccination.

## Discussion

**Figure 1.**
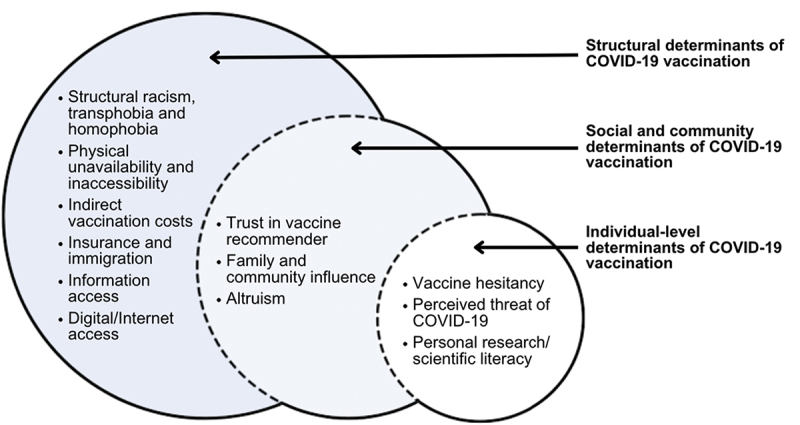
Multilevel determinants of COVID-19 vaccination among marginalized sexual and gender minority adults.

This study is, to our knowledge, the only in-depth qualitative exploration of COVID-19 undervaccination and vaccine hesitancy focused on the perspectives and experiences of racialized sexual and gender minority adults, populations occupying multiple marginalized demographic intersections that result in heightened vulnerability to COVID-19 transmission and severe outcomes. The spectrum of COVID-19 vaccine-related concerns and challenges, and interconnections among individual-, social- and structural-level phenomena, add complexity to understanding vaccine decision-making and uptake in the context of marginalization (see Figure 1).

Systemic barriers in access to vaccination were evidenced due to structural transphobia and homophobia, similar to findings from limited research in the U.S.^43,44^ Transgender and gender diverse individuals’ risk-benefit considerations of COVID-19 vaccination had to account for painful experiences of being deadnamed^42,45^ and otherwise not having their gender identity respected or acknowledged in the largely unfamiliar venues providing COVID-19 vaccination. In addition to health concerns due to disruptions in access to gender-affirming hormones in the pandemic, as reported in previous studies,^45,46^ and heightened vaccine-related safety concerns,^26^ the present findings highlight barriers in vaccination access due to structural transphobia. This indicates the need for gender-affirming approaches^46,47^ in the context of COVID-19 vaccination, including mitigation of risks to accessing vaccination and respectful engagement with transgender people’s vaccine-related concerns.

An intersectional approach to understanding the experiences of racialized sexual and gender minority individuals^48^ regarding COVID-19 vaccination provides further evidence of the impacts of marginalization on undervaccination. In addition to histories of medical racism, the legacy of electroconvulsive ‘therapy’ and diagnostic classification of “homosexuality” as a mental illness, along with ongoing albeit widely discredited practices of “conversion therapy,” perpetrate harm against sexual and gender minority individuals under the guise of healthcare and science.^49,50^ Beyond exposing challenges owing to structural homophobia, this may help to decouple “vaccine hesitancy” from Black and Latinx populations, as if a feature of racialized individuals rather than a function of social structures and institutions.^51^

Structural determinants of vaccine uptake were also evidenced in constraints to availability due to systemic disparities in access to healthcare by race/ethnicity and income.^52,53^ Participants reported overcrowding and months-long wait-times for vaccination appointments in the limited hospitals in some of the highest risk neighborhoods in the GTHA, and a smaller number of “pop-up” clinics in areas with the highest densities of community of color, as corroborated by media accounts at the time.^54^ Superimposed on these infrastructural inequities, challenges emerged in lack of access to clear and culturally tailored information about COVID-19 vaccination, especially in languages other than English, in a city in which 42.5% report a first language other than English or French.^55^ Constraints to digital/broadband internet access and digital literacy, particularly for older racialized adults, rendered sign-ups for limited vaccination appointments a monumental task, extending evidence about broadband Internet access as a social determinant of health^56^ to COVID-19 vaccination.

Importantly, while Canada affords basic healthcare to all its citizens, a variety of indirect costs presented barriers to vaccination. Costs of transportation to distant vaccination sites, lack of paid sick days amid daylong vaccination wait-times, and childcare needs rendered a vaccine out of reach for many from economically marginalized populations, often those disproportionately impacted by pandemic lockdowns, job layoffs, and housing insecurity.^57^

At the social and community level, both families of origin and families of choice provided support to individuals marginalized from the social and economic mainstream, promoting vaccine confidence through communication with trusted others. This suggests the importance of COVID-19 vaccine education and information campaigns, including traditional and social media, adopting an inclusive conceptualization of family in the context of the relationships and living situations of sexually and gender diverse, and racially and ethnically diverse populations, which may differ from those of White, cisgender, and heterosexual people.^58,59^

Notably, prominent positive influences on attitudes toward COVID-19 vaccination emerged in altruistic motivations on behalf of one’s family and community, as has been identified in the case of other vaccines.^60,61^ An overriding motivation to protect one’s community was evoked in the context of awareness of heightened vulnerability to COVID-19 infection and adverse health outcomes, and disruptions in employment, housing, and access to healthcare. This suggests that approaches to promoting COVID-19 vaccination among racialized and sexual/gender minority populations consider appeals to communitarian interests, and that they carefully weigh the risks of vaccine mandates, which may undermine altruistic motivations.^62,63^

In sum, the broader structural and social contexts that emerged as foundational to the COVID-19 vaccination experiences and perspectives of individuals occupying intersectional marginalized identities support the need for critical assessment and reconceptualization of “vaccine hesitancy”^14,28,29^ and trust in vaccination.^64^ Nevertheless, much of the burgeoning research on COVID-19 vaccine hesitancy among racialized populations appears to elide the latter part of the definition: “delay in acceptance or refusal of vaccination *despite the availability of vaccination services*” ^17(p.4163)^ [emphasis added]. The present findings suggest that various models of vaccine hesitancy, largely based on White cisgender heterosexual populations and parents’ vaccination of their children, risk de-emphasizing potent structural barriers to vaccination, particularly when extrapolated to marginalized populations.^28^ Structural barriers that were highly influential in vaccine uptake and decision-making in the present study risk being distilled and sanitized under “convenience,”^65^ with conceptualizations of vaccine “confidence” divorced from structural racism. As a result, interventions based on such models may be constrained in their effectiveness in promoting vaccination among marginalized populations.

At the individual level, this study provides evidence for COVID-19 vaccine hesitancy in the context of structural constraints. Participants presented an array of concerns in their decision-making about whether or not, and when, to be vaccinated, and who they would consider trustworthy sources of recommendation, and evoked what may be distinct aspects of vaccine hesitancy among intersectionally marginalized populations. As structural racism, transphobia and homophobia are unlikely to be eradicated before the next public health emergency, it is vital to recalibrate conceptualizations of vaccine hesitancy, and its operationalization in research, practice, and policy, to effectively support the decision-making processes of individuals from racialized and sexual/gender minority communities – often those with the highest vulnerability to infection and adverse outcomes.

Finally, the present findings suggest that effective responses to COVID-19 undervaccination among racialized sexual and gender minority communities should focus on developing and implementing interventions to eliminate multifaceted social-structural barriers in access to vaccination; identifying and mobilizing trusted sources of vaccine recommendations; and designing outreach strategies to disseminate community-relevant information to address various permutations of decisional ambivalence, conflict, mistrust, and resistance that may constitute vaccine hesitancy. These multilevel efforts should be informed by community histories of unethical medical experimentation, mistreatment and trauma, and ongoing experiences of structural racism,^66–68^ structural transphobia and homophobia.^27,33^

### Limitations and strengths

Findings should be understood in the context of study limitations. First, while it is not the objective of qualitative research to generalize, the few gay-identified men in this study may not capture the range of experiences among this population. Nevertheless, this study is unique in that research on sexual minority populations generally under-represents cisgender women and racialized individuals. The substantial number of transgender and gender diverse participants in this study is a further strength, enabling attention to issues specific to gender identity. Second, we purposefully included a range of participant self-identifications in recognition of racial and ethnic diversity in the adoption of sexuality and gender identity labels; however, we acknowledge the problematical use of “two-spirit” by non-indigenous people to denote gender identity or sexual orientation. Finally, while the successful recruitment of individuals from underrepresented populations immediately following pandemic restrictions was enabled by conducting study recruitment and data collection online, this may have favored the inclusion of younger people. Nevertheless, our findings also reflect concerns expressed by older adults, as well as those reported by younger participants about their parents and community members; these suggest that the structural barriers identified may be even more protracted among older adults.

## Conclusion

This study highlights pervasive barriers in access to and availability of COVID-19 vaccination among sexual/gender minority and racialized populations in the context of economic marginalization, structural racism,^69,70^ cis-sexism and heterosexism^27,43^ that confer inaccessibility to health innovations.^71^ Structural stigma and institutional mistrust produced by healthcare systems, government and public health establishments, and the pharmaceutical industry permeated the context of introduction and dissemination of a novel vaccine in a public health emergency. We also identified a spectrum of vaccine hesitancy^17^ reflected in “a state of indecision and uncertainty about vaccination before a decision is made to act (or not act),” ^72(p.58)^ that was integrally linked to structural determinants of health.^28,51,68,73^ This supports the importance of future research that examines and disaggregates structural determinants of vaccine availability, access, and inequity, from individual attitudes, beliefs, and decisional ambivalence that may constitute vaccine hesitancy.^51,73^ Otherwise, we risk reductionistic approaches that distill potent structural determinants of health through the lens of individual-level psychological factors. Vaccine hesitancy is thereby applied as a catch-all term in which undervaccination is understood as a “cultural barrier” characteristic of populations that endure marginalization, rather than approached as a structural barrier characteristic of systems and ideologies that perpetrate marginalization. This in effect places the onus of societal and institutional processes of marginalization, and undervaccination, on those disenfranchised by structural racism, transphobia, and homophobia.^33,51–53^

In fact, more prominent than COVID-19 “vaccine hesitancy” among individuals from marginalized communities, the present study identified “vaccine hunting”: taking individual and collective action to navigate a myriad of structural barriers in order to gain access to COVID-19 vaccination for oneself, one’s family and community in the face of ongoing health and broader structural inequities. Moving forward, community-engaged research and interventions are needed both to explore and address culturally informed understanding of vaccine hesitancy, and to effect changes in healthcare systems, policies, and practices to promote vaccine equity and access among individuals from sexual and gender minority and racialized populations.

## Supplementary Material

Supplemental file 1.docx
