# Supplementary material for: “Going vaccine hunting”: Multilevel influences on COVID-19 vaccination among racialized sexual and gender minority adults—a qualitative study
Source: Hum Vaccin Immunother. 2024 Feb 12;20(1):2301189. doi: 10.1080/21645515.2023.2301189 (PMC10863362; doi:10.1080/21645515.2023.2301189)
Supplement: Supplemental file 1.docx [file KHVI_A_2301189_SM9072.docx]

**Semi-structured interview guide**

- Can you tell me some of what was going on for you during the COVID-19 pandemic?
  - Probe: Can you describe some of the challenges you faced considering factors like gender, race, class differences, citizenship status, or other intersectional experiences?
  - Probe: any barriers to primary health care?
  - Probe: any barriers to gender-affirming healthcare?
- During the past year or so, COVID-19 vaccines became available as a way to protect oneself. Please share your thoughts on the COVID-19 vaccine.
  - Probe: You are free to answer or not, but have you been vaccinated or not?
  - Probe: How many doses have you had?
- What are your thoughts on specific barriers to COVID-19 vaccination affecting your community/communities?
- What factors of the vaccine itself are/were most important to you in making your decision about vaccination?
- Who would you trust the most to recommend the vaccine to you and why?
- If we were to have another pandemic, what supports and services would you hope we would have in place for vaccine distribution?
- Is there anything else you want to share about your experience with the pandemic?
